# Reflecting strategic and conforming gendered experiences of community health workers using photovoice in rural Wakiso district, Uganda

**DOI:** 10.1186/s12960-018-0306-8

**Published:** 2018-08-22

**Authors:** David Musoke, Charles Ssemugabo, Rawlance Ndejjo, Elizabeth Ekirapa-Kiracho, Asha S. George

**Affiliations:** 1 Department of Disease Control and Environmental Health, School of Public Health, Makerere University College of Health Sciences, Kampala, Uganda; 2 Department of Health Policy, Planning and Management, School of Public Health, Makerere University College of Health Sciences, Kampala, Uganda; 3 University of the Western Cape, Cape Town, South Africa

**Keywords:** Community health workers, Photovoice, Roles, Gender, Males, Females, Health systems, Uganda

## Abstract

**Background:**

Community health workers (CHWs) are an important human resource in Uganda as they are the first contact of the population with the health system. Understanding gendered roles of CHWs is important in establishing how they influence their performance and relationships in communities. This paper explores the differential roles of male and female CHWs in rural Wakiso district, Uganda, using photovoice, an innovative community-based participatory research approach.

**Methods:**

We trained ten CHWs (five males and five females) on key concepts about gender and photovoice. The CHWs took photographs for 5 months on their gender-related roles which were discussed in monthly meetings. The discussions from the meetings were recorded, transcribed, and translated to English, and emerging data were analysed using content analysis in Atlas ti version 6.0.15.

**Results:**

Although responsibilities were the same for both male and female CHWs, they reported that in practice, CHWs were predominantly involved in different types of work depending on their gender. Social norms led to men being more comfortable seeking care from male CHWs and females turning to female CHWs. Due to their privileged ownership and access to motorcycles, male CHWs were noted to be able to assist patients faster with referrals to facilities during health emergencies, cover larger geographic distances during community mobilization activities, and take up supervisory responsibilities. Due to the gendered division of labour in communities, male CHWs were also observed to be more involved in manual work such as cleaning wells. The gendered division of labour also reinforced female caregiving roles related to child care, and also made female CHWs more available to address local problems.

**Conclusions:**

CHWs reflected both strategic and conformist gendered implications of their community work. The differing roles and perspectives about the nature of male and female CHWs while performing their roles should be considered while designing and implementing CHW programmes, without further retrenching gender inequalities or norms.

## Background

Community health workers (CHWs) play a very important role in strengthening health systems and increasing availability of community-level primary health care services [1, 2]. Indeed, they are now regarded as an integral part of the health system and support the work of formal health professionals in many countries [2], including Uganda [3]. In Uganda, the Ministry of Health (MOH) introduced CHWs locally referred to as village health teams (VHTs) as the first point of contact for healthcare delivery in the community. The VHT strategy was intended to enhance the capacity to mobilize individuals and households for better health [4, 5]. CHWs in Uganda, majority of whom are female, comprise of community volunteers who can read and write preferably in their local language, selected by local leaders and trained by health professionals to provide accurate health information and appropriate linkages to health services. Specifically, CHWs carry out health education, conduct household visits to promote sanitation and hygiene, mobilize the community for public health interventions such as immunization, treat children below 5 years of age under integrated community case management of childhood illnesses (iCCM), and refer patients to health facilities.

Gender—the social roles, activities, characteristics, and behaviours that society prescribes for men and women—is an important dimension in human resources for health that has not been given due attention, especially in low- and middle-income countries [6–9]. With regard to CHWs, studies have shown that female CHWs can be deployed to services closely intertwined with gendered beliefs such as looking after children [10, 11], may not get recognized as skilled workers [12], can lack household support, and also may be affected by personal security concerns [13]. On the other hand, male CHWs usually have household support, receive recognition for their work, and are more respected [6]. While performance of CHWs in undertaking maternal and newborn work is not necessarily different by gender, community norms can make male and female CHWs differentially accepted [14]. Although recognition of gendered experiences of CHWs is emerging [7], very little is known about CHWs’ perspectives of such experiences using community-based participatory research methodologies such as photovoice.

Photovoice is a process by which people can identify, represent, and enhance their community through a specific photographic technique [15]. Its purposes include enabling people to record and reflect on their community’s strengths and concerns, promoting critical dialogue and knowledge about important community issues through large and small group discussion of photographs, and enabling communities to visually communicate with policy makers [15]. Photovoice can support empowerment and emphasizes individual and community strengths, co-learning, community capacity building, and balancing research and action [16]. It can enable people with limited power to capture aspects of their environment and experiences and share them with others [17]. All these strengths of the photovoice methodology were explored and reflected in our study.

Use of photovoice among CHWs can foster dialogue among themselves and community members about their experiences, facilitate CHW engagement in the research process, and enable their voice to be heard among various stakeholders including policy makers [15, 17, 18]. Understanding gender perspectives of CHWs themselves using photovoice is important to guide measures to address gender inequalities, and inform policy decisions regarding their work. In our study, we explore the differential roles of male and female CHWs in rural Wakiso district, Uganda, using photovoice, an innovative community-based participatory research approach.

## Methods

### Study design

The study, conducted in 2015 and 2016, was qualitative and used photovoice as a community-based participatory research method. The study employed ten CHWs (five males and five females) who took photographs concerning gender and ethics issues related to their work for a period of 5 months. This paper specifically presents the findings regarding gender from the research. During the period of photography, five monthly meetings were held among the participants and researchers to discuss the photos, and take note of emerging issues.

### Study area and setting

The study was conducted in Bulwanyi parish, Ssisa sub-county, Wakiso district, located in the central region of Uganda. Bulwanyi parish was purposively selected for involvement in this study due to the earlier work done by the researchers in the area [19]. Ssisa sub-county is predominantly rural with a population of 94 238 (45 272 males and 47 966 females), 23 992 households, and an average household size of 3.8 [20]. The main economic activities carried out in the area are agriculture and small-scale trade. A section of the population is also involved in brick making and stone quarrying. As is the case in most parts of the country, CHWs exist in this area and are the first contact of the community with the health system.

### Selection of study participants

The ten CHWs who participated in the research were selected by local leaders in the area who included village chairpersons and mobilizers. These local leaders did the selection because they were familiar with the CHWs that existed in the community. The criteria used in the selection of CHWs were provided by the researchers which included having an equal gender representation (five males and five females) and diversity in terms of socio-economic status and village of origin. The CHWs were selected from various villages so as to expand the range of experiences during photography and subsequent discussions. The selected CHWs were aged 25 to 55 years, and among them, nine had attended secondary education while eight were married with an average of six children (Table 1). All the CHWs had been involved in this role of supporting their communities for over 3 years.

**Table 1:** Demographics of participants

Participant number	Gender	Age	Village	Education level	Main occupation	Marital status and number of children
1.	Male	55	Bulwanyi	Primary	Agriculture	Married, 8 children
2.	Female	30	Lukose	Secondary (ordinary level)	Agriculture	Married, 5 children
3.	Female	49	Kaama	Secondary (ordinary level)	Agriculture	Widowed, 7 children
4.	Male	48	Kaama	Secondary (ordinary level)	Agriculture	Married, 8 children
5.	Male	40	Bumpenje	Secondary (ordinary level)	Agriculture	Married, 5 children
6.	Female	47	Lukose	Secondary (ordinary level)	Agriculture	Married, 7 children
7.	Female	50	Bumpenje	Secondary (ordinary level)	Agriculture	Married, 5 children
8.	Female	28	Kaama	Secondary (ordinary level)	Business	Married, 4 children
9.	Male	25	Bulwanyi	Secondary (advanced level)	Agriculture	Single, none
10.	Male	31	Lukose	Secondary (advanced level)	Agriculture	Married, 3 children

### Training workshop

After selecting the participants, a training workshop was conducted to establish the CHWs’ understanding of gender and ethics in their own perspective and to orient them on the research on gender and ethics using photovoice. The workshop, which was conducted at a field office of Makerere University School of Public Health in Ssisa sub-county, was facilitated by the researchers and lasted 1 day. The topics covered during the training were photovoice methodology, research principles such as voluntary participation and informed consent, CHWs’ perspectives on gender including differential roles of male and female CHWs, use and care for cameras, and ethical issues in photovoice research. During the training, every CHW was provided with a digital camera for use during the research. In addition, CHWs were provided with a notebook to take note of any issues related to the research that may not be captured on camera for example when consent to take photos is not provided by a community member.

### Photography assignment

At the end of the training workshop, CHWs were assigned to take photographs related to gender and ethics during the course of their work for a period of 5 months. The CHWs were not restricted on the number of photos they could take as long as they related to the study themes of gender and ethics. A follow-up meeting of CHWs by the research team 2 weeks after commencement of photography was conducted so as to address the challenges faced during taking photographs such as those related to charging the cameras. During the entire period of photography, CHWs were supervised by the researchers to ensure the assignment was carried out as planned, and to address any emerging challenges. The supervision involved regular visits to the CHWs as well as providing support needed as part of the research.

### Discussing photographs and data analysis

All photos taken by CHWs for purposes of the study were presented and discussed in five monthly meetings. In addition to CHWs, the meetings which lasted between 2 and 3 h were facilitated by two researchers. One researcher facilitated the meetings while the other provided support including note taking and logistical assistance such as transferring photos to a laptop. The meetings for the first 3 months were conducted separately for males and females while those for the last 2 months were held jointly. The separation of males and females was done to allow free discussion of gender-related issues. The last two meetings were combined so as to explore emerging issues that required the presence of CHWs of both genders. During these meetings, each CHW talked about the photographs they took and how they related to the study topics. The photographs, which were transferred to the laptop before commencement of the meeting, were projected to a screen to facilitate the discussion. After each photo had been presented, other CHWs had the opportunity to talk about it either asking a question or discussing it in another way they felt related to the study themes. After exhausting all photos a CHW had taken in the previous month, they were given the opportunity to present any issues that they had captured in their notebook. Any issues that emerged from the previous monthly meeting were presented after all photographs had been discussed.

All proceedings of the meetings were audio recorded in the local language (*Luganda*) and later transcribed verbatim by one of the researchers. After verification of the transcripts, they were translated to English for analysis. The translated transcripts were read by another member of the research team to ensure that the activity had been done adequately. The transcripts were imported into Atlas ti version 6.0.15 and read several times to identify initial codes based on repeated and emerging issues. Data were analysed using conventional content analysis [21] where codes and categories arising from the data were used. During the analysis, researchers immersed themselves in the data without any preconceived categories allowing new insights to emerge. Related codes identified from the data were grouped together to form categories and later merged into themes. All themes are described in the results and illustrated with selected quotes and photographs from CHWs.

### Ethical consideration

The study got approval from Makerere University School of Public Health Higher Degrees, Research, and Ethics Committee, and was registered at the Uganda National Council for Science and Technology. All CHWs provided voluntary written informed consent before participation in the study after clearly explaining to them the nature of the research including potential risks and benefits. It was emphasized to the CHWs during the training that they needed to obtain verbal consent from community members before they took their photographs. No personal information about the CHWs was collected during course of the study, and all research data were handled confidentially. No photograph taken as part of the study was to be used for any form of dissemination (including this publication) without the written consent of both the photographer and any individual(s) appearing in it.

## Results

A total of 432 photographs were taken during the study yielding an average of eight per CHW per month. The results are presented under seven main themes as follows: addressing men on their terms; female-related health issues; treatment of children; response to emergencies; geographic coverage during community mobilization; involvement in manual work; and availability to offer services in community.

### Addressing men on their terms

From the photos and resulting discussions, it was noted that male CHWs were consulted more by men for their own health concerns. These concerns included personal illness, condom use, circumcision, and other reproductive health issues. They also consulted them about other issues that were not directly related to reproductive health such as alcoholism. Men’s preference for male CHWs is captured in the quotation below.

Men easily explain their private sexuality issues to us [male CHWs] for example when they contract sexually transmitted infections, they can come and describe to us the symptoms they are experiencing and we advise them accordingly. In addition, they also find it easier to request for condoms from male CHWs which is sometimes harder for them to do among female CHWs. (Photographer 1, male, age 55)

**Fig. 1:**
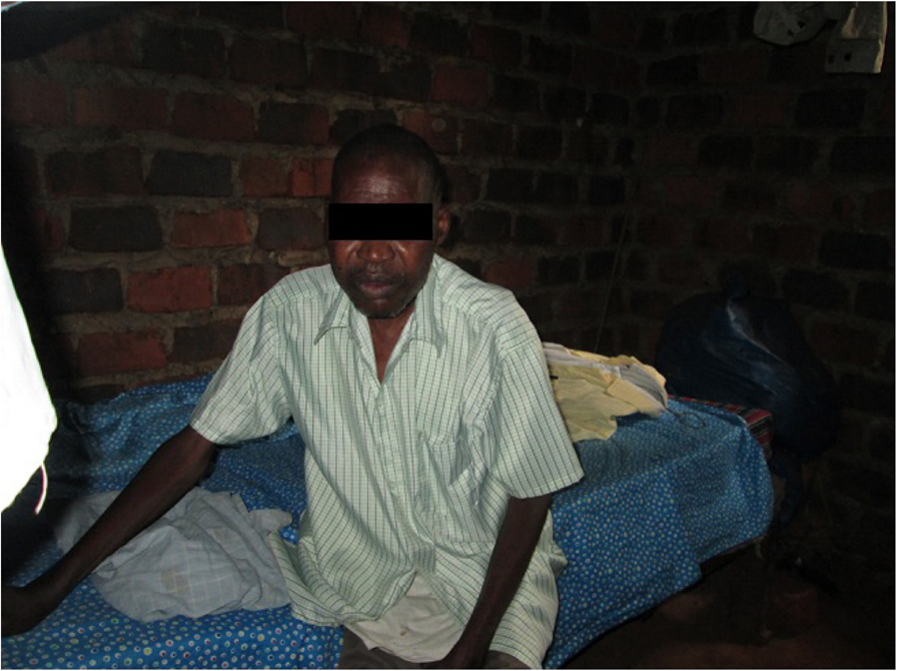
A sick man who had called a male CHW to his bedroom to attend to his condition

When men in the community fall sick, they were quick to contact male CHWs. This was established to be partly because they would not want to reveal personal information to female CHWs including knowing about their health condition. Indeed, in scenarios where men had conditions which required physical body examination, male CHWs were more utilized (Fig. 1).

This man was sick and in his bedroom. He invited me to his home to attend to him. Being male, it was easier for me to go to his bedroom and examine him from there which may not have been the case with a female CHW. He easily removed his shirt and explained to me his illness, and I later referred him to the health facility. (Photographer 5, male, age 40)

In some cases, women in the community who had family problems contacted male CHWs to counsel their husbands. These family issues included failure to provide adequate support to their families, refusal to escort their wives to the hospital, and denial of using contraception which highlights the potentially transformatory role male CHWs can play in changing gender inequalities as illustrated below.

This lady was pregnant and she requested me to speak to the man responsible for her pregnancy to tell him the importance of seeking antenatal care as she had wanted to start going to the health facility but had not received support from him. She told me that she had tried to speak to him herself but the man refused to listen, and that since I am a male CHW, the man would easily heed my advice. I then met the man and told him to go to the health facility with his wife which he accepted. I later met the lady and she was so happy. She told me that she had gone with him to the health centre and they were attended to very fast therefore she was very thankful for the role I played. (Photographer 5, male, age 40)

### Female-related health issues

CHW discussions spurred by the photographs also highlighted that when women had reproductive health issues such as concerning family planning, female CHWs were preferred and easily accepted. Female CHWs were also often called upon to counsel teenagers and married couples in the community, at times resorting to gender-conforming coping strategies, as shown below.

There is a woman who had quarreled with the husband who later beat her so much yet she was 6 months pregnant. She then came and told me about it, and asked for advice because she wanted to leave the man. As a female CHW, who is married, and a parent, it was easy for her to approach me, and I told her to calm down and talk to the husband, because she wanted to leave the children yet it would be bad to leave them there without a mother. (Photographer 2, female, age 30)

From the study discussions, female CHWs were more knowledgeable on maternal issues including pregnancy danger signs and antenatal care. They had a lot of experience in dealing with most of the maternal health problems which they often shared with women. CHWs revealed that women in the community confide a lot in female CHWs especially when they are faced with pregnancy-related problems. Female CHWs also usually paid visits to expectant women and advised them accordingly.

### Treatment of children

**Fig. 2:**
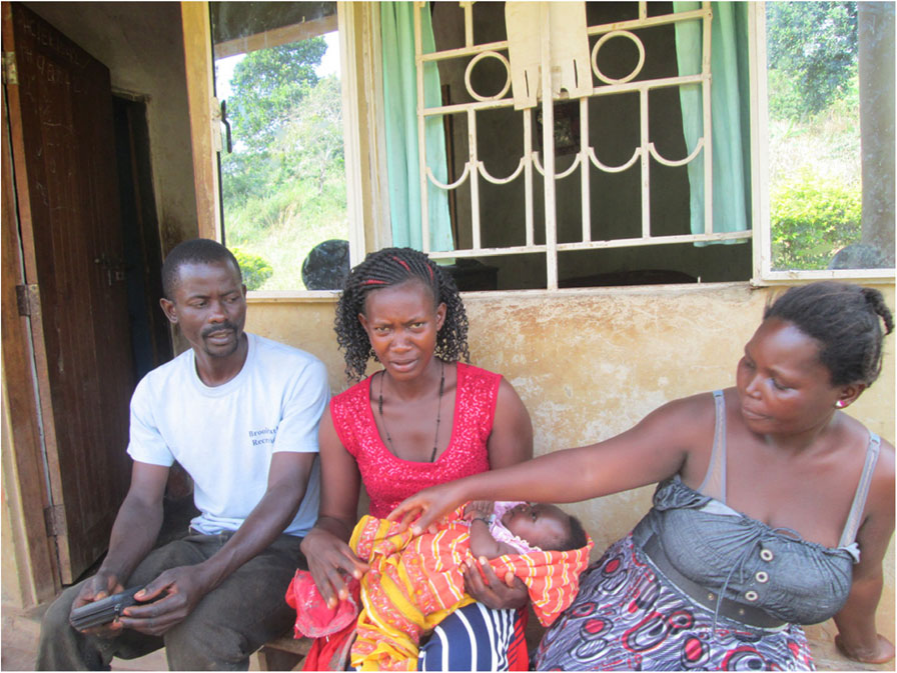
A female CHW (centre) visiting a couple that had a baby

It was noted that although both male and female CHWs treat childhood illnesses, females were more involved in this activity, and other related roles such as immunization. It emerged from the discussions and photos that female CHWs spent more time treating children and exhibited a lot of passion while handling them (Fig. 2). Experiences of female CHWs looking after their own children contributed to their comfort in supporting children and the confidence community members had in them. Indeed, community members were many times keen to seek services from female CHWs whenever their children were sick or in need of a public health intervention as illustrated below.

In that photo, that woman had refused to take the child for immunisation. I approached her and told her to take the child to be immunised as it was being carried out by qualified health professionals. I explained to her well and she agreed to take the child for immunisation. She easily accepted what I had told her since I am a mother with children just like her. (Photographer 2, female, age 30)

### Response to emergencies

**Fig. 3:**
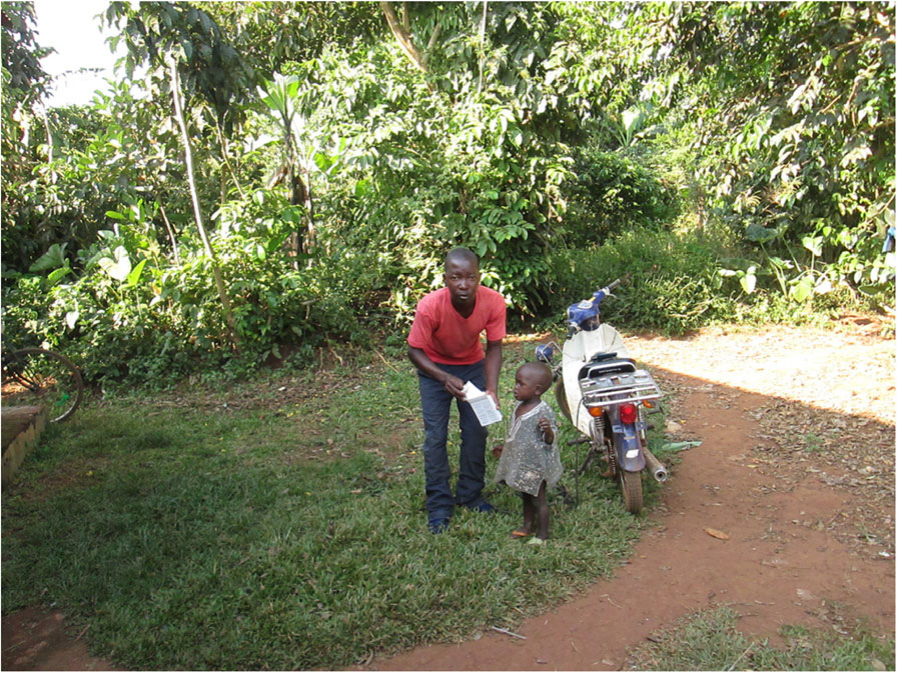
A male CHW prepares to take a sick child to the health facility on his motorcycle

It was revealed from the photos and discussions that male CHWs were faster in responding to health emergencies in terms of taking the sick, especially children and pregnant women, to health facilities than their female counterparts. Ease of male CHWs responding to emergencies was because they had more access to means of transport such as bicycles and motorcycles (Fig. 3). This was not only in terms of possession but also the social norms that constrain women’s ability to ride bicycles or motorcycles.

Men find it easier than women to rush sick children to the hospital in case they are referred. In that photo, the child was sick and I had referred him to a health facility. As a male CHW who owns a motorcycle, I managed to quickly take the child to the health facility. (Photographer 9, male, age 25)

As a male CHW, I can use any means of transport available like a bicycle or motorcycle or car to transport a patient to a health facility in case of any problem in the community. For a female CHW, it might be hard to use certain means of transport like bicycles to transport a patient to the hospital. (Photographer 10, male, age 31)

### Geographic coverage during community mobilization

Regarding mobilising communities for public health campaigns such as immunization or monitoring field activities across villages, CHWs agreed that male CHWs covered larger areas compared to females. Male CHWs’ advantages to use transport also enabled them to take up supervisory roles within CHWs’ work which include data collection and compilation among fellow CHWs.

“When we are doing our roles in the community like visiting homes, men travel longer distances since we are more energetic than women. A man can move to a larger area and visit many homes if the day is for conducting home visits compared to a woman. (Photographer 10, male, age 31)

### Involvement in manual work

**Fig. 4:**
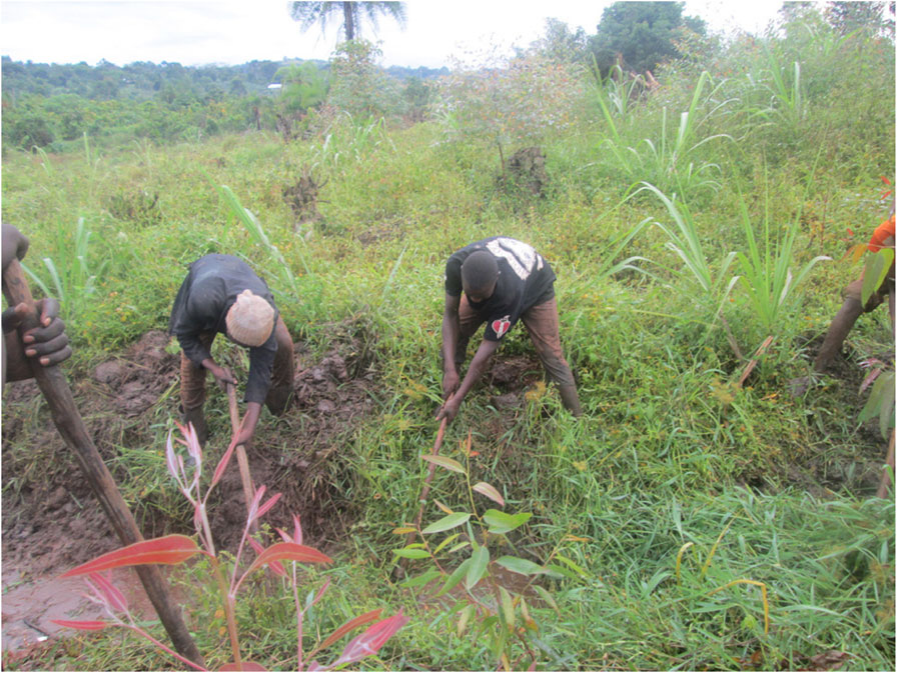
A male CHW (left) with a colleague digging a drainage channel for a community water source

The photos and discussions indicate that male CHWs were more involved in manual activities such as renovation of latrines, cleaning and desilting wells (Fig. 4), and constructing barriers to prevent animals from accessing water sources. This was because of the gendered division of labour in communities in addition to social norms that restrict women from engaging in physical interventions such as cleaning wells. Instead, women were engaged in other activities like mobilising the community, digging the path to the sources, and preparing food for the men who were working.

I was trying to help a certain woman since her latrine wall was cracked and the bricks had fallen off on one side so I was putting them back. This was necessary as she was being seen by passersby whenever she was using the latrine. However, if I was a female CHW, I may not have managed to put back those bricks as it involved a lot of energy. (Photographer 9, male, age 25)

### Availability to offer services in community

**Fig. 5:**
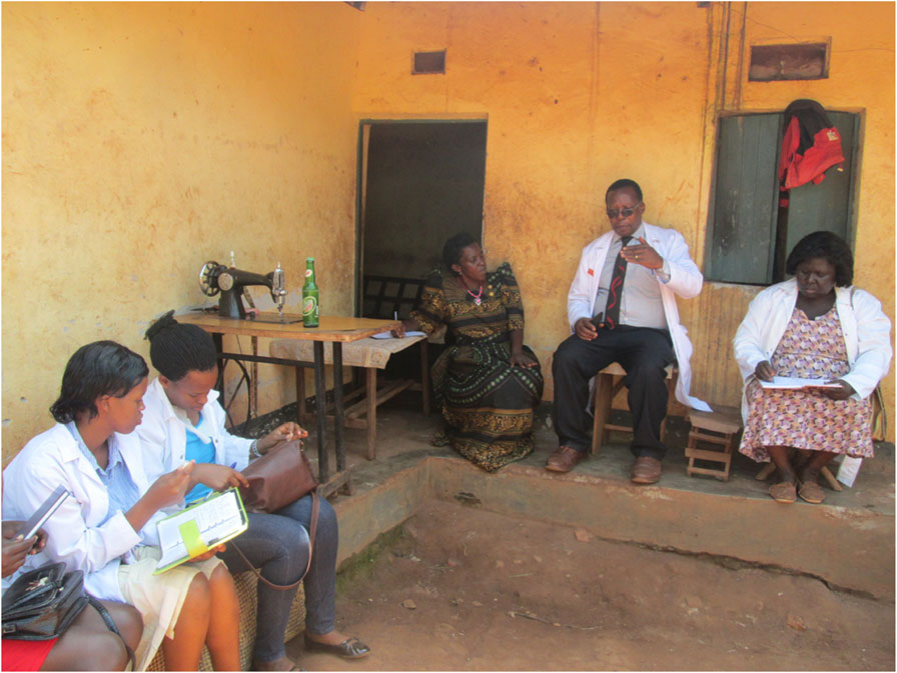
A female CHW (centre) sharing her experiences with a team from a university during a students’ field attachment activity

From the study discussions, it was established that female CHWs spent more time in the communities due to the gendered division of paid labour. Women spend most of their time at home doing unremunerated domestic and care work such as cooking, house cleaning, washing clothes, and looking after children. The few female CHWs who carried out income-generating activities were either involved in agriculture in their home gardens or small-scale retail business within their homes. Therefore, anytime there was a problem or a health visitor in the community (Fig. 5), female CHWs were usually locally available to attend to them, in contrast to male CHWs who often went for income-generating work away from their homes and communities.

I was treating a patient very early in the morning. It is sometimes hard to find a male community health worker at home at such a time. Many times, they would have already gone for work but me, I was still around. (Photographer 8, female, age 28)

**Fig. 6:**
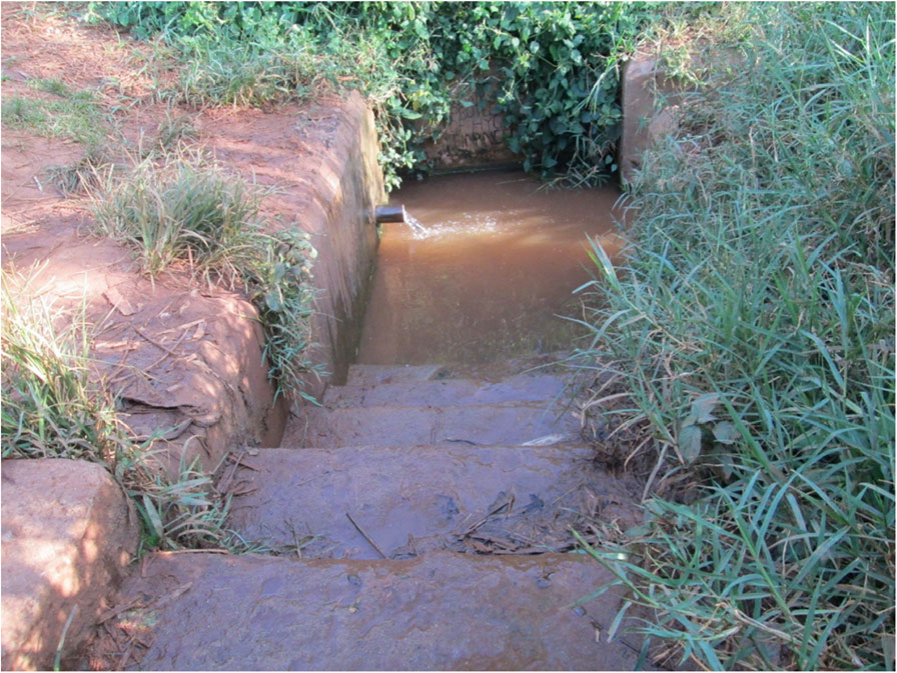
A poorly drained water source in the community identified by a female CHW

Since female CHWs were based in their communities, they were also able to identify and discuss with other community members issues related to water, sanitation, and hygiene. These include poorly drained wells (Fig. 6), people washing near water sources, unscreened latrines, and latrines without superstructure.

I found these children washing clothes near the water source as I had gone to collect water. I asked them why they were doing so and they said that it is very hard for them to fetch water to take home for washing clothes. I asked who had sent them and they told me that it was their parents. I took the responsibility and went to speak to the parents, and explained to them the implications this can have, and we agreed that this would not happen again. It is usually females who go to wells hence it is easier for them to find such concerns in the community*.* (Photographer 3, female, age 49)

## Discussion

Our photovoice study provides insights into various aspects of the gendered experience of CHWs in rural communities in Uganda, reflecting strategic positioning, gendered access to resources, division of labour, community values, and social norms. Whereas female CHWs are in larger numbers, engage more in treating children, and are more available to offer services in the community due to the gendered division of labour, male CHWs are more able to respond faster to emergencies due to more access to transportation means, cover larger areas during mobilization, enlist in supervisory work, and carry out more manual activities. These findings reveal potentially differing roles for male and female CHWs while supporting primary health care and public health in rural communities in Uganda.

From our exploratory study, CHWs felt that community members are generally more likely to consult a CHW of the same gender concerning health issues. This finding is understandable as a CHW of the same gender as a community member would not only use their expert knowledge on the issue of concern while attending to them but also their personal experiences, as shown in other settings [14]. It has also been shown even among health professionals that patients many times prefer to see those of the same gender as them [22–24]. Nevertheless, for scenarios that are not intimate and psychosocial, patients may opt to seek health care from a health worker of the opposite gender [25]. Moreover, in some settings such as in Brazil, female CHWs were not seen to be able to contest conservative gender social norms around male responsibility for sexually transmitted infections and gender-based violence [10]. Having CHWs of both genders in the community may ensure that members can seek health care from CHWs of their preference hence increasing utilization of the volunteer services, but also provide partnerships to strategically revisit gender norms.

One of the main responsibilities of CHWs is to provide treatment to children under 5 years of age suffering from malaria, diarrhoea, and pneumonia under integrated community case management of childhood illnesses (iCCM). It was established from our exploratory study that although male CHWs also treated children, female CHWs were perceived to be more involved in this role. Experiences of female CHWs looking after their own children could be a contributor to their role in management of childhood illnesses. As CHW programmes scale up, they require higher educational levels, entail handling more medical commodities, and become more remunerated, and male CHWs can take a dominant role despite policy intent to the contrary [26]. Given that CHWs play a significant role in primary health care [27, 28], understanding their gendered experiences is important in ensuring that health systems as a part of the social fabric of societies act as mechanisms that promote gender equality, rather than retrench gender conformity.

Although male CHWs were only 25% in the study area, our research established that they are involved in certain roles more than their female counterparts. Male CHWs were faster in responding to emergencies, covered larger areas in the community during mobilization, and were more involved in manual work such as repairing faulty sanitation facilities. Increased ownership and utilization of transport means by male CHWs helped them in taking referred patients to health facilities. This role of facilitating transportation of patients is very important due to the severe transportation challenges faced particularly in rural areas in Uganda [29–31]. Without quick response to urgent issues such as rushing a patient to a health facility, health outcomes for example maternal and child mortality are likely to be worse. Mobilising communities for interventions such as mass immunization, in which male CHWs are more involved due to access to resources and social norms among other favouring factors, is also crucial to ensure high coverage of public health initiatives. Due to gendered roles of CHWs, communities without male CHWs may face limitations in carrying out such activities unless these gendered barriers and norms against female CHWs are addressed.

The work of CHWs is voluntary in many countries including in Uganda where they do not receive any remuneration for their work. As men are expected to be the main income earners for families in rural settings, they are less willing to be involved in voluntary work as their prime identity [32, 33]. The few existing male CHWs may only be involved in CHW roles after carrying out their income-generating activities. This gender-related responsibility of men providing for their families is important to appreciate in the work of CHWs as it affects their availability in the community. In our study, we established that due to the gendered division of labour and because female labour is often not remunerated, female CHWs were more available to offer services in the community in comparison to males. Many female CHWs stay at home, where they are involved in agriculture, and hence can be found in case health care is needed. It is important to balance this finding of women’s increased availability to offer services given their marginalized roles in rural economies and guard against health systems exploiting women’s availability as a form of cheap labour [34].

A strength of this paper is that it presents gendered perspectives of CHWs as seen by them during the course of their work. Indeed, photovoice as a community-based participatory research methodology enables participants to take the lead in the data collection (photography) activity and express their viewpoints more directly as was the case in our study. However, given that our exploratory study was carried out in one rural parish in Wakiso district, the findings may not be generalizable to a larger or contextually different geographical setting. There is also a need for further research on community perspectives regarding gendered access and acceptability of CHWs which we did not explore. Nevertheless, our study provides important information on gendered roles of CHWs in a rural setting in Uganda. In addition, our experiences of using the photovoice methodology can inform future studies in Uganda and other parts of the world.

## Conclusions

CHWs reflected both strategic and conformist gendered implications of their community work. As a participatory methodology, photovoice lends itself to revealing gendered community values and can be a starting point for addressing gendered inequalities in communities. The differing roles and viewpoints about the nature of male and female CHWs during the course of their work should be considered while designing and implementing CHW programmes, without further retrenching gender inequalities and norms. This would ensure that not only CHWs are effective in their roles as health agents and equality advocates but also health systems more broadly serve to address gender inequalities and advance health outcomes equitably.

## Abbreviations

CHW: Community health worker
iCCM: Integrated community case management of childhood illnesses
VHT: Village health team
